# Effect of exercise intervention on smoking cessation: a meta-analysis

**DOI:** 10.3389/fphys.2023.1221898

**Published:** 2023-08-08

**Authors:** Yuehui Zhou, Wenxia Feng, Yugang Guo, Juhua Wu

**Affiliations:** ^1^ School of Sport Science, Qufu Normal University, Qufu, Shandong, China; ^2^ School of Physical Education, Anyang Normal University, Anyang, Henan, China; ^3^ School of Sport, Guangxi University of Science and Technology, Liuzhou, Guangxi, China

**Keywords:** effect, exercise, meta-analysis, smoking cessation, mood, tobacco dependence, cravings

## Abstract

**Background:** Exercise has emerged as an effective approach to promote individual health and has shown potential in aiding smoking cessation. However, the specific benefits of exercise in smoking cessation remain unclear, and conflicting findings across studies may be attributed to variations in study populations and intervention characteristics. This study aims to conduct a meta-analysis to evaluate the impact of exercise interventions on tobacco dependence in smokers and assess the effectiveness of exercise in facilitating smoking cessation.

**Methods:** A comprehensive search was performed in databases including PubMed, Web of Science, Embase, The Cochrane Library, and Scopus to identify relevant randomized controlled trials published before 30 October 2022. The Preferred Reporting Items for Systematic Review and Meta-Analyses (PRISMA) guidelines were followed during the review process. The quality of evidence (QoE) was assessed with GRADE (grading of recommendations, assessment, development and evaluations) methodology.

**Results:** Acute exercise was found to significantly reduce smoking cravings [MD = −1.84, 95% CI (−2.92, −0.76), *p* < 0.001; SMD = −1.64, 95% CI (−2.22, −1.05), *p* < 0.001] and alleviate most withdrawal symptoms in smokers. However, there was no significant difference in the smoking cessation rate between the exercise group and the control group (*p* > 0.05). Exercise was associated with increased positive mood [SMD = 0.36, 95% CI (0.14, 0.58), *p* = 0.001] and reduced negative mood in smokers [SMD = −0.26, 95% CI (−0.39, −0.12), *p* < 0.001].

**Conclusion:** Acute exercise interventions effectively reduce cravings and withdrawal symptoms in smokers. However, long-term exercise interventions do not significantly improve the smoking cessation rate. Exercise can help reduce negative mood and enhance positive mood in smokers. Smokers with high levels of tobacco dependence may derive less benefit from exercise. Factors such as literature quality, exercise intervention characteristics, and exercise adherence may influence the effectiveness of interventions.

**Trial registration:** This research protocol was registered in the International Prospective Register for Systematic Reviews (PROSPERO https://www.crd.york.ac.uk/PROSPERO/). Registration number: CRD42022326109.

## 1 Introduction

Smoking remains one of the leading preventable causes of premature death worldwide, resulting in approximately 8 million deaths annually (World Health Organization, 2021). The “China Smoking Health Report 2020” released by China’s National Health Commission revealed that China has over 300 million smokers, with a smoking rate of 26.6% among individuals aged 15 years and older (Council, 2019). Smoking is a significant contributing factor to various diseases, including lung cancer, chronic respiratory diseases, coronary heart disease, stroke, and diabetes (West, 2017). Recent studies have also demonstrated that smokers face a significantly higher risk of COVID-19 progression and mortality compared to nonsmokers (Li et al., 2021). The “Health China Action (2019–2030)” issued by China’s National Health and Wellness Commission aims to reduce the smoking prevalence among individuals aged 15 years and older to less than 24.5% by 2022 and 20% by 2030 (Opinions of the State Council on the Implementation of Health China Action, 2019). Despite the implementation of effective tobacco control measures such as policy bans, health education, and medical consultations, quitting smoking remains challenging due to the highly addictive nature of nicotine, psychological and behavioral habits, and sociocultural factors associated with tobacco use. Approximately 75%–95% of smokers relapse within 6 months of attempting to quit (Livingstone-Banks et al., 2019). Therefore, it is crucial to actively explore effective strategies to reduce tobacco dependence.

Previous interventions to address tobacco dependence have primarily focused on psychological interventions, self-management techniques, and medication. Although these approaches demonstrate relatively significant short-term effects, their long-term efficacy is limited, with success rates ranging from only 7%–9% (Hughes et al., 2014). Moreover, self-management and psychological interventions require specific psychological skills that smokers often find difficult to navigate on their own (García-Gómez et al., 2019), Medication, on the other hand, may lead to side effects and potential dependence (Batra et al., 2016).

In recent years, with the increasing focus on the relationship between exercise and health, the role of exercise in tobacco dependence cessation has garnered significant attention. Exercise has been proposed as a standalone or adjunctive treatment for smoking cessation due to its potential to alleviate withdrawal symptoms, cigarette cravings, concerns about weight gain, as well as improve mood and mitigate the adverse effects of smoking on cardiorespiratory function (Haasova et al., 2014; Klinsophon et al., 2017). However, the findings from academic research on the effects of exercise interventions on tobacco dependence have been inconclusive, particularly regarding the long-term effects of such interventions (Ho et al., 2014; Ussher et al., 2019). A review of recent meta-analyses in the field of exercise interventions for smoking cessation has identified the following key characteristics: 1) Many studies lacked strict inclusion criteria. For instance, pregnant women (Klinsophon et al., 2017) and individuals with psychiatric disorders (Santos et al., 2021) were not consistently excluded, and literature on exercise counseling as an intervention was not consistently excluded either. Considering that physiological differences during pregnancy may affect nicotine withdrawal symptoms, including changes in hormone levels, which may affect nicotine metabolism and withdrawal response s (Míguez et al., 2019). In addition, pregnant women typically have higher motivation to quit smoking than non-pregnant adults because they have to consider not only their health but also the health of their fetus (Tong et al., 2008; Miyazaki et al., 2013; Patnode et al., 2021; Jackson et al., 2022). These may affect their willingness and success in trying to quit smoking. Therefore, Pregnant women were excluded from this study. Individuals with mental illness often experience unstable mental health, which can make smoking cessation or reduction particularly challenging (Schöttl et al., 2022). The inclusion of these studies may have influenced the overall results of the meta-analysis. 2) The use of a single outcome measure makes it difficult to obtain a comprehensive understanding of the effects of exercise (Klinsophon et al., 2017; Santos et al., 2021). 3) Most studies did not investigate potential moderating variables between exercise and tobacco dependence, such as exercise intervention characteristics. These variables are crucial in the development of effective intervention programs, and their absence leads to inadequate guidance for existing exercise programs. Furthermore, the search deadline for the most recent reviews in this field was January 2018, and several new randomized controlled trials have been published since then. Therefore, a new review is necessary to build upon the observations from previous meta-analyses and incorporate the latest evidence. In this study, a comprehensive set of outcome measures was employed, and the literature was screened more rigorously. Subgroup analysis was conducted to identify potential factors that influence the effects of exercise interventions, aiming to achieve a more comprehensive understanding of the effects of exercise. Meta-analysis was employed to systematically review existing studies and clarify the impact of exercise on tobacco dependence among smokers.

## 2 Materials and methods

The review was conducted in accordance with the PRISMA (Preferred Reporting Items for Systematic Reviews and Meta-Analyses) guidelines (Page et al., 2021), and the protocol had previously been registered in PROSPERO (CRD42022326109). It is important to note that the statement “screening literature pre-dates registration” in the study registration form was an error made by the registrant. The official search for this study commenced on 30 October 2022. We want to emphasize that our study was not biased as a result of this error.

### 2.1 Search strategy

Randomized controlled trials (RCTs) of exercise interventions for smoking cessation were searched in the PubMed, Web of Science, Embase, The Cochrane Library, and Scopus databases. The search period ranged from the inception of each database to 30 October 2022. Additional sources, including previously published reviews, gray literature, expert documents, reference lists of eligible studies, and relevant reviews, were also searched. The search employed a combination of subject terms and free words. YH was responsible for identifying search terms and developing search strategies, while WX executed the specific search implementation. The search terms encompassed topics such as smoking, tobacco, nicotine, cigarettes, exercise, sport, physical activity, and randomized controlled trial. To illustrate, Table 1 presents the specific search strategy used for the PubMed database.

**TABLE 1:** *PubMed* database retrieval strategy.

Search	Query
#1	exercise [MeSH]
#2	sport [MeSH]
#3	physical activity [MeSH]
#4	#1 OR #2 OR #3
#5	smoke [MeSH]
#6	smoking [MeSH]
#7	smokers [MeSH]
#8	nicotine [MeSH]
#9	tobacco [MeSH]
#10	cigarette [MeSH]
#11	smoking cessation [MeSH]
#12	#5 OR #6 OR #7 OR #8 OR #9 OR #10 OR #11
#13	animal study
#14	randomized controlled trial [All Fields]
#15	#4 AND #12 NOT #13 AND #14

### 2.2 Inclusion and exclusion criteria

#### 2.2.1 Inclusion criteria

Participant Type: We included individuals who were dependent on tobacco, without any restrictions on age or gender.

Design Type: This analysis included randomized controlled trials (RCTs).

Intervention Types: We included interventions aimed at increasing exercise, either as a standalone approach or as an adjunct to smoking cessation interventions. These were compared with smoking cessation programs alone or other non-exercise control groups. The control and exercise groups differed in their interventions only in whether or not they exercised, e.g., the control group intervention was nicotine replacement therapy and the exercise group intervention was exercise + nicotine replacement therapy.

Outcome Types: The study considered several outcome indicators, including craving, withdrawal symptoms, smoking cessation rate, and mood. The trials reported at least one of these available data.

#### 2.2.2 Exclusion criteria

The exclusion criteria were as follows:

(1) Studies involving patients with concurrent substance dependence other than tobacco (e.g., alcohol, drugs, etc.), pregnant women, or individuals with psychiatric disorders.
(2) Studies without a control group or with a control group that included an exercise intervention.
(3) Studies with incomplete or unquantified data for outcome indicators or lack of appropriate outcome indicators.
(4) Qualitative studies, reviews, case studies, animal studies, and duplicate publications.
(5) Articles for which the full text was not accessible through various channels.

### 2.3 Study selection and data extraction

Literature search records were managed using EndNote X8 software (Clarivate Analytics, Philadelphia, PA, United States). The results of the database searches were imported and combined with EndNote X8, and duplicates were removed. Two researchers (YH and WX) independently screened the study titles and abstracts, retrieved and assessed the full text for compliance with inclusion criteria. Any discrepancies were resolved through discussion with YG if necessary, until a consensus was reached. Once the screening was complete, the full text was reviewed again, and data extraction was performed. Two researchers (YH and WX) independently extracted and entered the data. YG intervened to review and verify the data in cases of disagreement or inconsistency. The extracted information included three aspects (World Health Organization, 2021): basic information about the included literature, such as the first author, year of publication, characteristics of the study population (sample size, age, sex, tobacco dependence), content of the interventions in the experimental and control groups, intervention protocol (intensity, duration, frequency, period, etc.), and outcome indicators used (Council, 2019); information on the quality evaluation of the included literature; and (West, 2017) data indicators included in the literature, such as means and standard deviations of pre- and post-tests for each outcome and the number of events. If figures were reported graphically without providing the required data, GetData Graph Digitizer software (version 2.22) was used for data extraction.

### 2.4 Quality assessment

The Cochrane Risk of Bias tool was utilized to assess the quality of the included RCTs based on seven indicators: method of random allocation, allocation concealment, blinding of participants (and personnel), blinding of outcome assessment, completeness of outcome data, selective reporting of study results, and other sources of bias. The quality of the study was categorized into 3 grades: high (low risk for 4 or more entries), moderate (low risk for 2 or 3 entries), and low (low risk for 1 or no entries, potential for bias) (Wu et al., 2017). Risk of bias assessment was performed independently by two review authors (YH and WX), and any disagreements were resolved through discussion with a third author (YG).

### 2.5 Data analysis

Data were collated and analyzed using RevMan software version 5.4 and Stata software version 16.0. For continuous outcomes such as craving and withdrawal symptoms, weighted mean differences (WMDs) or standardized mean differences (SMDs) with 95% confidence intervals (CIs) were used as effect sizes. WMD was estimated when outcome measurements across all studies used the same scale, while SMD was employed when outcomes were measured using different quantitative scales (Higgins et al., 2019). Reported effect sizes were classified as trivial (<0.2), small (0.2 to <0.5), moderate (0.5 to <0.8), and large (≥0.8) (Cohen, 2013). For dichotomous outcomes, including adverse events, relative risks (RRs) with 95% CIs were pooled. *I* ^2^ was used to test the heterogeneity of the included studies, in which 25, 50% and 75% of the *I* ^2^ value were the judgment thresholds of low, medium and high heterogeneity, respectively (Higgins et al., 2003). Fixed-effects models were employed when heterogeneity was low; otherwise, random-effects models were used for the analysis. Sensitivity analysis was conducted by excluding trials with an assessed risk of bias to test the robustness of the pooled results. Exploratory subgroup analyses were performed to examine whether various factors influenced the effect size estimates. Publication bias tests could be conducted using funnel plots and quantified using Egger’s method (Sterne et al., 2011). For the evaluation of the quality of evidence (QoE), the GRADE methodology was used, evaluating five domains: inconsistency, risk of bias, imprecision, indirectness, and publication bias. Finally, QoE was presented in summary tables (SoF) using GRADEpro GDT (https://gradepro.org/, accessed on 12 July 2023. Any decisions to downgrade the certainty of studies were justified in footnotes.

## 3 Results

### 3.1 Literature selection

**FIGURE 1:**
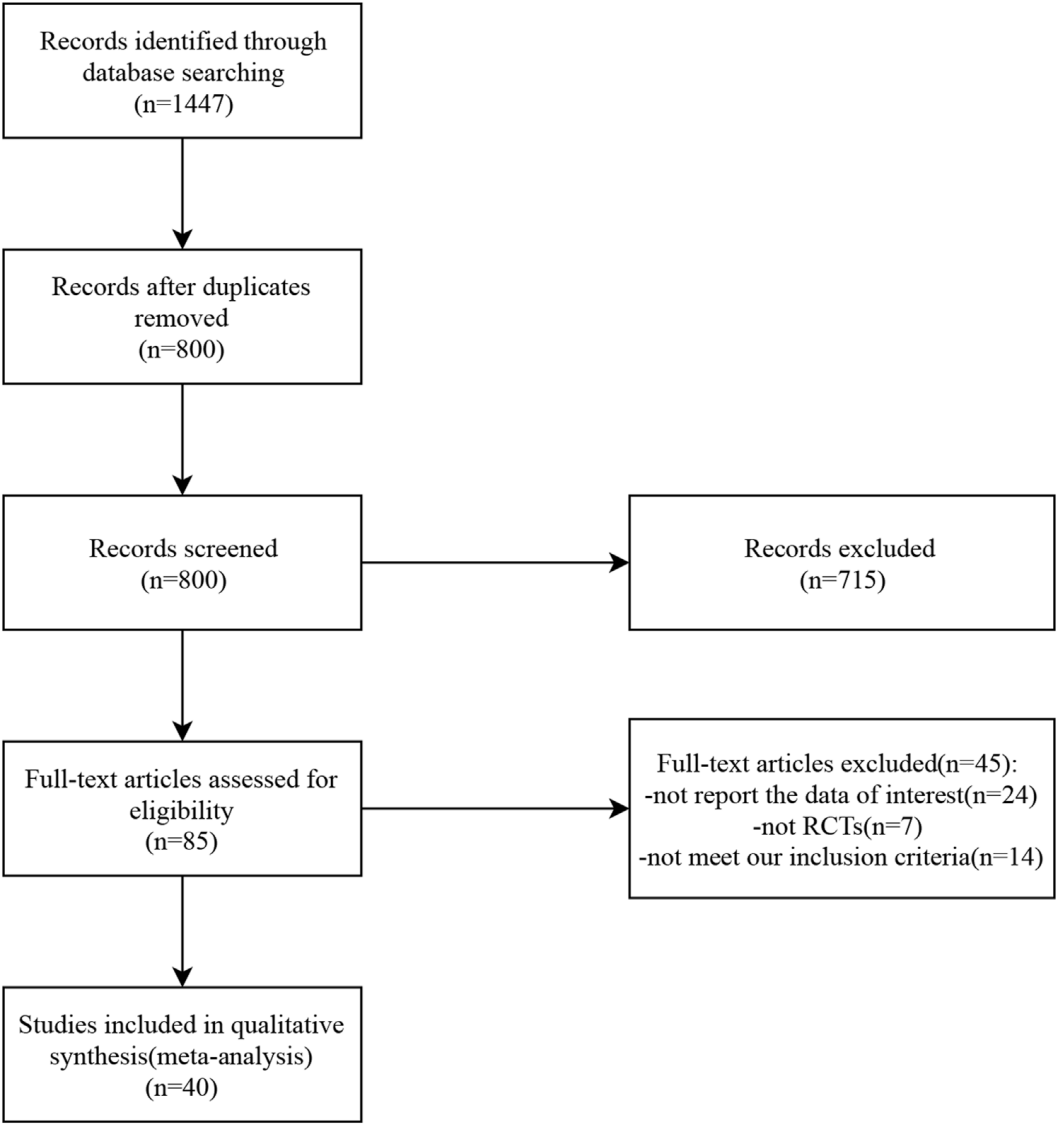
Screening flow diagram.

A total of 1,447 studies related to the topic of this study were retrieved from seven databases. After eliminating duplicates, 800 studies remained. The titles and abstracts of these studies were reviewed, and 85 studies were selected for further assessment. Among them, 45 studies were excluded: 24 did not report relevant data, seven were not randomized controlled trials (RCTs), and 14 did not meet the inclusion criteria. Finally, 40 studies were included in the analysis. The detailed selection process is presented in Figure 1.

### 3.2 Characteristics of the included studies

Table 2 provides an overview of the included studies. The 40 studies encompassed a total of 43 RCTs, with three studies comprising 2 RCTs each [denoted as (World Health Organization, 2021; Council, 2019), respectively]. The meta-analysis included a total of 3,427 smokers. Ten studies exclusively enrolled female participants, while the remaining studies included both sexes. The age range of participants in the included studies was 18–65 years.

**TABLE 2:** Characteristics of the included studies.

Author	Sample (M/F)	Age (Y)	Degree of nicotine dependence	Exercise schedule					Control group intervention	Outcome indicator
				Type	Minutes per session	Frequency	Duration	Intensity		
Ussher et al. (2001)	78 (36/42)	36.6 ± 10.9	6.1 ± 2.3	Stationary cycling	10 min	-	-	40%–60% HRR	Sitting passively, watch video	B_1_, B_2_
Ussher et al. (2006)	60 (33/27)	32.19 ± 8.94	3.9 ± 2.12	Isometric exercise	5 min	-	-	-	Sitting passively	B_2_, C
Ussher et al. (2009)	48 (31/17)	27.8 ± 8.4	5.0 ± 2.2	Isometric exercise	10 min	-	-	-	Read, body scanning	B_2_, C
Taylor et al. (2005)	15 (10/5)	25.6 ± 6.5	4.0 ± 3.1	Self-paced walking	15–20 min	-	-	RPE 10.8 ± 1.49	Sitting passively	B_2_
Taylor et al. (2006)	15 (10/5)	25.6 ± 6.5	4.0 ± 3.1	Walking on treadmill	15–20 min	-	-	RPE 10.8 ± 1.49	Sitting passively	B_1_
Taylor and Katomeri (2007)	60 (26/34)	28.3 ± 7.4	3.47 ± 2.23	Walk on treadmill	15 min	-	-	RPE 10.9 ± 1.4	Sitting passively	B_1_, B_2_, C
Van Rensburg and Taylor (2008)	23 (15/8)	23.1 ± 4.6	3.4 ± 2.03	Walk on treadmill	15 min	-	-	RPE 10.8 ± 1.67	Sitting passively	B_1_
Van Rensburg et al. (2009a)	20 (15/5)	29.05 ± 9.37	4.0 ± 2.5	Stationary cycling	15 min	-	-	RPE 11–13	Sitting passively	B_1_
Van Rensburg et al. (2009b)	10 (6/4)	18–50	3.4 ± 1.6	Stationary cycling	10 min	-	-	RPE 11–13	Sitting passively	B_1_
Van Rensburg et al. (2012)	20 (−)	18–50	2.3 ± 1.3	Stationary cycling	10 min	-	-	RPE 11–13	Sitting passively	B_1_, B_2_
Van Rensburg et al. (2013)	162 (107/55)	30.8 ± 9.8	4.8 ± 1.9	Treadmill exercise	20 min	-	-	(1) 40% HRR	Watch video	D
								(2) 75% HRR		
Faulkner et al. (2010)	18 (10/8)	24.6 ± 5.9	4.5 ± 2.3	brisk walking	10 min	-	-	RPE 11.89 ± 1.79	Sitting passively	B_1_
Everson et al. (2008)	45 (25/20)	21.8 ± 2.2	3.36 ± 1.89	Cycling	10 mim	-	-	(1) 40–59%HRR	Sitting passively	B_2_, C
								(2) 60–84%HRR		
Oh and Taylor (2014)	23 (15/8)	23.96 ± 4.83	2.78 ± 1.78	Cycling	15 min	-	-	(1) 40%–50% HRR	Sitting passively	B_2_
								(2) 70%-75%HRR		
Tritter et al. (2015)	30 (10/20)	40.19 ± 10.30	4.53 ± 2.27	Treadmill exercise	15 min	-	-	45%–68% HRR	Sitting quietly/Read	B_1_
Masiero et al. (2020)	50 (24/26)	23.83 ± 3.65	4.00 ± 1.41	Cycling	10 min	-	-	Moderate intensity		B_1_
Jeffries et al. (2020)	55 (34/21)	28.16 ± 10.4	2.98 ± 2.01	Yoga	30 min	-	-	-	Read	B_1_
Scerbo et al. (2010)	18 (10/8)	26.0 ± 4.2	4.4 ± 1.7	Walking/Running	15 min	-	-	(1) 45–50%HRR	Sitting passively	B_1_, B_2_
								(2) 80–85%HRR		
Schneider et al. (2015)	48 (14/34)	42.63 ± 13.38	4.22 ± 1.93	Treadmill exercise	10 min	-	-	40%–68% HRR	Sitting passively	B_1_
Fong et al. (2014)	25 (11/14)	37.5 ± 14.8	3.84 ± 2.36	Treadmill exercise	15 min	-	-	45%-68%HRR	Sitting passively	B_2_
De Jesus and Prapavessis (2018)	110 (56/54)	33.41 ± 14.13	4.61 ± 1.95	Treadmill exercise	10 min	-	-	40–68%HRR	Sitting passively	B_1_
Hill (1985)	36 (10/26)	25–50	-	Aerobic exercise	30 min	2times/wk	5 weeks	-	Group counselling	A_1_
Hill et al. (1993)	82 (39/43)	50+	6.5 ± 1.6	Aerobic exercise	15–35 min	1–3 times/wk	12 weeks	60%–70% HRR	Behavioral training	A_1_
Marcus et al. (1995)	20 (0/20)	37.5 ± 8.9	-	Aerobic exercise	30–45 min	3 times/wk	15 weeks	70%–85% HR_max_	Health education	A_1_
Marcus et al. (1999)	281 (0/281)	40.2 ± 8.96	6.1 ± 2.0	Aerobic exercise	30–40 min	3 times/wk	12 weeks	60%–85% HRR	Health education	A_1_, A_2_
Marcus et al. (2005)	217 (0/217)	42.77 ± 10.34	4.85 ± 2.32	Aerobic exercise	30–45 min	5 days/wk	8 weeks	45%–59% HRR	Health education	A_1_, A_2_
Prapavessis et al. (2007)	121 (0/121)	38.0 ± 11.7	-	Aerobic exercise	45 min	3 times/wk	12 weeks	60%–75% HR_max_	Health education	A_1_, A_2_
Al-Chalabi et al. (2008)	40 (19/21)	34.9 ± 11.7	5.2 ± 2.3	Isometric exercise	-	-	4weeks	-	Sitting passively	A_1_
Kinnunen et al. (2008)	182 (0/182)	38.4 ± 9.6	4.8 ± 2.3	Aerobic exercise	30 min	1–2 times/wk	19 weeks	60%–80% HR_max_	Health education	A_2_
Bize et al. (2010)	481 (272/209)	42.4 ± 9.7	5.4 ± 2.2	Aerobic exercise	45 min	1 time/wk	9 weeks	40–60%VO_2max_	Health education	A_2_, D
Williams et al. (2010)	60 (0/60)	42.37 ± 11.55	4.82 ± 2.52	Aerobic exercise	50 min	3times/wk	8 weeks	70% HR_max_	Watch films	A_1_, A_2_
Ciccolo et al. (2011)	25 (12/13)	36.5 ± 12.0	4.0 ± 2.6	Resistance exercise	60 min	2 times/wk	12 weeks	65%–75% RM	Watch video	A_1_, A_2_
Linke et al. (2012)	38 (15/23)	43.6 ± 11.5	5.2 ± 2.3	Multi-component exercise	60 min	-	12 weeks	RPE 12–14	Internet-based smoking cessation program	A_1_
Bock et al. (2012)	55 (0/55)	45.6 ± 8.3	5.0 ± 1.4	Yoga	45 min	2 times/wk	8 weeks	-	Watch video	A_1_, D
Whiteley et al. (2012)	330 (0/330)	43.52 ± 9.96	5.12 ± 2.12	Aerobic and resistance exercise	40–65 min	3 times/wk	12 weeks	64–85%HR_max_	Health education	A_1_, A_2_
Abrantes et al. (2014)	61 (21/40)	47.3 ± 9.6	5.7 ± 1.9	Aerobic exercise	20–30 min	2–4 times/wk	12 weeks	55%–69% HR_max_	Health education	A_1_, A_2_
Abrantes et al. (2018)	57 (18/39)	47.95 ± 9.18	5.85 ± 1.86	Aerobic exercise	15–40 min	2–4 times/wk	12 weeks	55%–69% HR_max_	Health education	D
Cheung et al. (2020)	208 (156/52)	40.2 ± 9.9	-	Isometric exercise	-	-	14 weeks	-	Health education	A_1_
Dunsiger et al. (2021)	105 (0/105)	42.5 ± 11.2	-	Aerobic exercise	50 min	3 times/wk	12 weeks	64%–76% HR_max_	View video	A_1_, A_2_
Oncken et al. (2020)	301 (0/301)	55.8 ± 6.2	5.3 ± 1.9	Aerobic and resistance exercise	60 min	2 times/wk	24 weeks	50%–69% HR_max_	Relaxation	A_1_

Abbreviations: M, male; F, female; Y, years; HRR, heart rate reserve; HR, heart rate; VO2 max, maximal oxygen consumption; A1, seven-day point quit rate; A2, sustained quit rate; B1, smoking craving; B2, intensity of smoking craving; C, withdrawal symptoms; D, mood.

Of the included studies, 21 focused on acute exercise interventions with a duration of 5–30 min (Ussher et al., 2001; Taylor et al., 2005; Taylor et al., 2006; Ussher et al., 2006; Taylor and Katomeri, 2007; Everson et al., 2008; Van Rensburg and Taylor, 2008; Van Rensburg et al., 2009a; Van Rensburg et al., 2009b; Ussher et al., 2009; Faulkner et al., 2010; Scerbo et al., 2010; Van Rensburg et al., 2012; Van Rensburg et al., 2013; Fong et al., 2014; Oh and Taylor, 2014; Schneider et al., 2015; Tritter et al., 2015; De Jesus and Prapavessis, 2018; Jeffries et al., 2020; Masiero et al., 2020). The remaining 19 studies involved long-term exercise interventions ranging from 4 to 19 weeks (Hill, 1985; Hill et al., 1993; Marcus et al., 1995; Marcus et al., 1999; Marcus et al., 2005; Prapavessis et al., 2007; Al-Chalabi et al., 2008; Kinnunen et al., 2008; Bize et al., 2010; Williams et al., 2010; Ciccolo et al., 2011; Bock et al., 2012; Linke et al., 2012; Whiteley et al., 2012; Abrantes et al., 2014; Abrantes et al., 2018; Cheung et al., 2020; Oncken et al., 2020; Dunsiger et al., 2021). The included studies incorporated various exercise modalities, including aerobic exercise, isometric exercise, resistance exercise, yoga, and multicomponent training (combinations of different exercises such as aerobic exercise, resistance exercise, balance, and flexibility exercises). Among these, aerobic exercise was the most frequently utilized, with cycling, walking, and treadmill activities being the predominant forms.

### 3.3 Results of risk of bias

**FIGURE 2:**
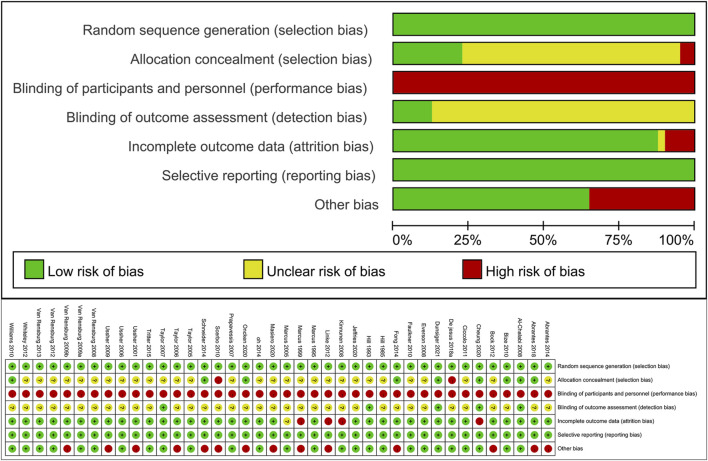
Evaluation of the risk of study bias for inclusion.

The assessment of risk of bias in the included trials is summarized in Figure 2. All 40 included studies employed random allocation and did not selectively report study outcomes. However, ensuring participant blinding was challenging due to the nature of exercise interventions, and most studies did not provide specific details on assessor blinding, resulting in a high risk of bias. Some of the included studies had missing outcome data, and although some studies had similar proportions of missing data between intervention groups or provided acceptable explanations, three studies still had a high risk of bias. Additionally, 12 (32%) studies reported other sources of bias, primarily including small sample sizes and significant baseline differences (*p* < 0.05). Based on the Cochrane Risk of Bias Assessment Tool, 7 studies were classified as low quality, 21 as moderate quality, and 12 as high quality. Overall, the methodological quality of the included studies was moderate.

### 3.4 Effects of acute exercise on smoking cravings

#### 3.4.1 Meta-analysis results and heterogeneity test

**FIGURE 3:**
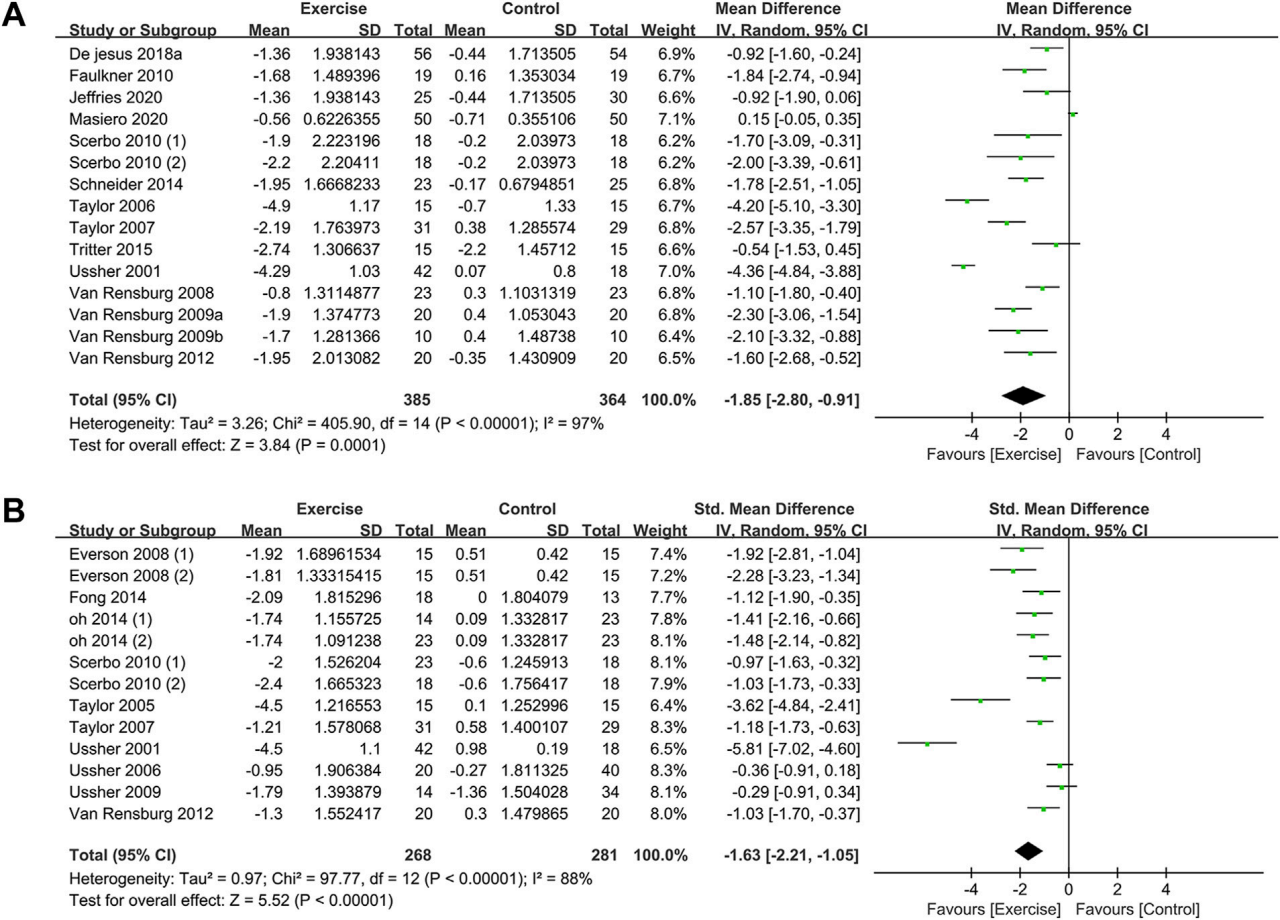
Meta-analysis of the effect of acute exercise on craving in tobacco -dependent individuals: **(A)** DtS and **(B)** SoD.

Smokers’ cravings were assessed using two Likert scales: desire to smoke (DtS) and strength of desire (SoD). A total of 15 RCTs (749 participants in total, with 385 in the exercise group and 364 in the control group) were included for DtS (Ussher et al., 2001; Taylor et al., 2006; Taylor and Katomeri, 2007; Van Rensburg and Taylor, 2008; Van Rensburg et al., 2009a; Van Rensburg et al., 2009b; Faulkner et al., 2010; Scerbo et al., 2010; Schneider et al., 2015; Tritter et al., 2015; De Jesus and Prapavessis, 2018; Jeffries et al., 2020; Masiero et al., 2020). Ten studies measuring SoD were included (Ussher et al., 2001; Taylor et al., 2005; Ussher et al., 2006; Taylor and Katomeri, 2007; Everson et al., 2008; Ussher et al., 2009; Scerbo et al., 2010; Van Rensburg et al., 2012; Fong et al., 2014; Oh and Taylor, 2014), involving a total of 554 participants (273 in the exercise group and 281 in the control group). The meta-analysis revealed that exercise interventions reduced DtS among smokers with a large effect size, based on a random-effects model [mean difference (MD) = −1.85, 95% confidence interval (CI) (−2.80, −0.91), *p* < 0.001]. The exercise interventions also had aa large effect size on SoD compared to the control group, as indicated by a standardized mean difference (SMD) of −1.63, 95% CI (−2.21, −1.05), *p* < 0.001 (Figure 3).

Heterogeneity tests showed high levels of heterogeneity for both DtS (*I* ^2^ = 97%, *p* < 0.001) and SoD (*I* ^2^ = 88%, *p* < 0.001). Sensitivity analysis, which involved removing individual studies, did not yield significant changes in the effect sizes for either outcome, indicating the robustness of the data.

#### 3.4.2 Subgroup analysis

Subgroup analyses were conducted to analyze exercise intervention characteristics, including exercise duration (≤10 min or >10 min) and exercise intensity (exercise intensity reported in the original studies prevailed; if not reported, the criteria in Table 3 for exercise intensity classification were applied). Since most of the included studies focused on aerobic exercise, the number of studies investigating other types of exercise was limited (less than two). Therefore, exercise types were not used as a covariate. The random-effects model was employed for the analysis. The subgroup analysis results for DtS indicated that (1) exercise duration of more than 10 min had a significant effect in reducing DtS [MD = −1.93, 95% CI (−2.76, −1.09), *p* < 0.001], whereas exercise durations of ≤10 min did not significantly reduce DtS [MD = −1.77, 95% CI (−3.62, 0.07), *p* = 0.059], and (Council, 2019) all exercise intensities had a significant effect on DtS. Notably, the high-intensity group had only one included study, and its results require further exploration.

**TABLE 3:** Exercise intensity grading.

Intensity	%HRmax	%VO2max	%HRR	%1-RM	RPE scale
Light	<64	<45	<40	<50	<11
Moderate	64 to <76	46 to <64	40 to <60	50 to <70	12–13
High	≥76	≥64	≥60	≥70	>13

For SoD, the subgroup analysis results showed that 1) the included RCTs focused on exercise durations of 10 min and 15 min, and both durations yielded significant effects, and (Council, 2019) three studies compared different exercise intensities in relation to SoD. Heterogeneity tests within each intensity group revealed substantial heterogeneity between studies. Thus, the random-effects model was used to estimate the effect sizes. The difference between the low-intensity group and the control group was not statistically significant [SMD = −2.40, 95% CI (−4.88, 0.07), *p* = 0.057], whereas both the medium-intensity group [SMD = −2.01, 95% CI (−3.11, −0.91), *p* < 0.001] and high-intensity group showed significant differences compared to the control group [SMD = −1.57, 95% CI (−2.24, −0.90), *p* < 0.001].

Subgroup analyses were performed to investigate the impact of various study characteristics on the effectiveness of the intervention, including literature quality (risk of bias categorized as low, medium, or high), sample size (≤50 or >50), and the level of tobacco dependence (FTND <4.5 or FTND ≥4.5) among participants. The analysis was conducted using a random-effects model. The results revealed the following findings (World Health Organization, 2021): Medium- and high-quality literature demonstrated more favorable effects of exercise interventions on both DtS and SoD among individuals with tobacco dependence compared to low-quality literature (Council, 2019). Sample size had an influence on the intervention effects. Studies with sample sizes greater than 50 showed less significant effects of exercise interventions on craving production [SMD = −1.72, 95% CI (−3.77, 0.32), *p* = 0.099; SMD = −2.40, 95% CI (−4.50, −0.11), *p* = 0.040] (West, 2017). Smokers with higher levels of tobacco dependence [SMD = −1.94, 95% CI (−3.96, 0.09), *p* = 0.061; SMD = −3.07, 95% CI (−8.55, 2.42), *p* = 0.273] were less likely to derive benefits from exercise compared to smokers with lower levels of tobacco dependence (refer to Table 4 for detailed results).

**TABLE 4:** Subgroup analysis of the effect of each factor on craving.

Outcome	Subgroup factors	Grouping criteria	Research number	Heterogeneity test results		Effect size [95%CI]	P
				I^2^	P		
DtS	Quality of the literature	Low	6	98.6	0.000	−2.37 [−4.61, −0.13]	0.038
		Medium	8	49.7	0.053	−1.39 [−1.81, −0.98]	0.000
		High	1	\	\	−2.57 [−3.35, −1.79]	0.000
	Sample size	<50	10	77.1	0.000	−1.92 [−-2.55, −1.29]	0.000
		≥50	5	98.7	0.000	−1.72 [−3.77, 0.32]	0.099
	Tobacco dependence Extent	FTND<4.5	11	94.7	0.000	−1.81 [−2.76, −0.87]	0.000
		FTND≥4.5	4	96.8	0.000	−1.94 [−3.96, 0.09]	0.061
	Exercise time	≤10min	7	98.1	0.000	−1.77 [−3.62, 0.07]	0.059
		>10min	8	84.5	0.000	−1.93 [−2.76, −1.09]	0.000
	Exercise intensity	Low	3	93.1	0.000	−2.61 [−4.33, −0.88]	0.003
		Medium	10	97.3	0.000	−1.70 [−3.93, −0.47]	0.007
		High	1			−2.00 [−3.39, −0.61]	0.005
SoD	Quality of the literature	Low	5	79.3	0.000	−1.98 [−3.74, −0.22]	0.028
		Medium	8	95.5	0.000	−1.61 [−222, −0.99]	0.000
		High	1	95.9	\	−1.20 [−1.75, −0.65]	0.000
	Sample size	<50	10	74.1	0.000	−1.46 [−1.94, −0.98]	0.000
		≥50	3	88.2	0.000	−2.40 [−4.50, −0.11]	0.04
	Tobacco dependence Extent	FTND<4.5	11	72.3	0.000	−1.42 [−1.84, −1.00]	0.000
		FTND≥4.5	2	98.5	0.000	−3.07 [−8.55, 2.42]	0.273
	Exercise time	10min	8	91.7	0.000	−1.70 [−2.60, −0.81]	0.000
		15min	5	76.1	0.002	−1.61 [−2.31, −0.92]	0.000
	Exercise intensity	Low	2	92.8	0.000	−2.40 [−4.88, 0.07]	0.057
		Medium	6	57.2	0.000	−2.01 [-3.11, −0.91]	0.000
		High	3	91.1	0.097	−1.57 [−2.24, −0.90]	0.000

#### 3.4.3 Publication bias test

Egger’s test was used for the publication bias test, and the results showed that SoD indicators might have publication bias (*p* < 0.05); Dts indicators were less likely to have publication bias (*p* > 0.05).

### 3.5 Effects of acute exercise on withdrawal symptoms

#### 3.5.1 Meta-analysis results and heterogeneity test

This study utilized six indicators to assess withdrawal symptoms: restlessness, irritability, tension, stress, poor concentration, and depression. The analysis included four studies with a total of 228 participants (Ussher et al., 2006; Taylor and Katomeri, 2007; Everson et al., 2008; Ussher et al., 2009), with 95 in the exercise group and 133 in the control group. The meta-analysis revealed that exercise interventions were more effective than the control group in alleviating withdrawal symptoms, except for stress, with both medium and small effect sizes.

**FIGURE 4:**
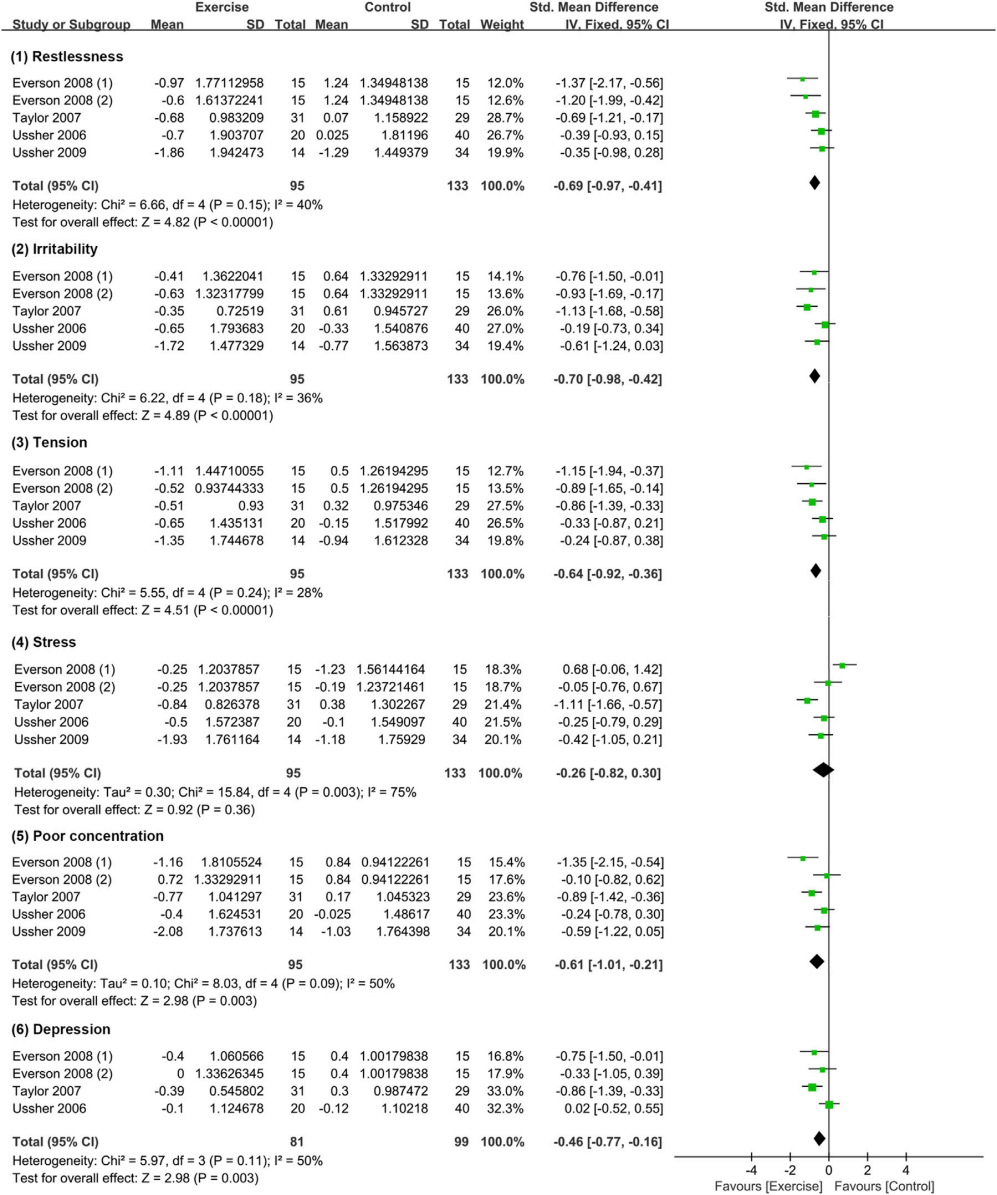
Meta-analysis of the effect of acute exercise on withdrawal symptoms in tobacco-dependent individuals.

The heterogeneity test indicated that restlessness (*I* ^2^ = 40%, *p* = 0.640), irritability (I2 = 36%, *p* = 0.180), tension (*I* ^2^ = 28%, *p* = 0.240), and depression (*I* ^2^ = 50%, *p* = 0.110) had moderate heterogeneity, whereas stress (*I* ^2^ = 75%, *p* = 0.003) and poor concentration (*I* ^2^ = 50%, *p* = 0.090) exhibited substantial level of heterogeneity. Due to the small sample size, meta-regression and subgroup analysis could not be conducted to identify the sources of heterogeneity. However, through sensitivity analysis, two studies, namely, Taylor and Katomeri, (2007) and Everson et al., (2008), were identified as sources of heterogeneity, and their exclusion resulted in heterogeneity reduced to 0 for both indicators (refer to Figure 4).

### 3.6 Effect of long-term exercise on smoking cessation rates

#### 3.6.1 Meta-analysis results and heterogeneity test

**FIGURE 5:**
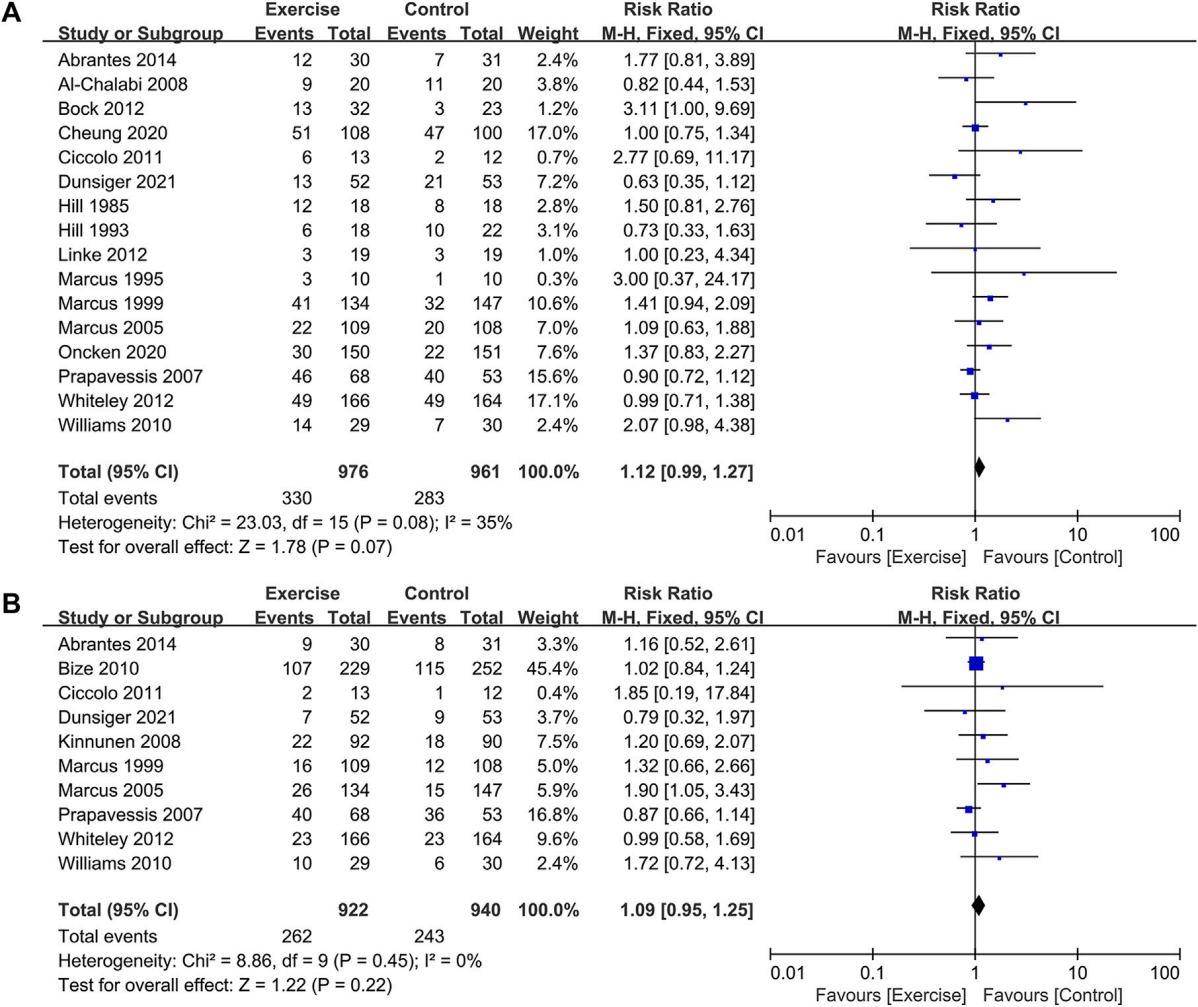
Effect of long-term exercise on smoking cessation rates in tobacco-dependent individuals: **(A)** 7-day point prevalence abstinence and **(B)** continuous abstinence.

The analysis included a total of 16 randomized controlled trials (RCTs) involving 1937 participants, with 976 in the exercise group and 961 in the control group, to assess the 7-day point prevalence abstinence as the outcome (Hill, 1985; Hill et al., 1993; Marcus et al., 1995; Marcus et al., 1999; Marcus et al., 2005; Prapavessis et al., 2007; Al-Chalabi et al., 2008; Williams et al., 2010; Ciccolo et al., 2011; Bock et al., 2012; Linke et al., 2012; Whiteley et al., 2012; Abrantes et al., 2014; Cheung et al., 2020; Oncken et al., 2020; Dunsiger et al., 2021). Additionally, 10 RCTs comprising 1862 participants, with 922 in the exercise group and 940 in the control group, were included to evaluate continuous abstinence (Marcus et al., 1999; Marcus et al., 2005; Prapavessis et al., 2007; Kinnunen et al., 2008; Bize et al., 2010; Williams et al., 2010; Ciccolo et al., 2011; Whiteley et al., 2012; Abrantes et al., 2014; Dunsiger et al., 2021). The meta-analysis found no significant difference in the change of 7-day point prevalence abstinence [RR = 1.12, 95% CI (0.99, 1.27), *p* = 0.080] and continuous abstinence [RR = 1.09, 95% CI (0.95, 1.25), *p* = 0.220] between the exercise group and the control group. This suggests that long-term exercise interventions did not significantly enhance smoking cessation rates. As the seven-day point prevalence indicator for smoking cessation is moderate level of heterogeneity (*I* ^2^ = 35%, *p* = 0.070) and the sustained point prevalence indicator for smoking cessation is low level of heterogeneity (*I* ^2^ = 0%, *p* = 0.450), a fixed-effects model was employed for the analysis. Sensitivity analysis conducted for each indicator by excluding individual studies demonstrated no significant change in effect size or heterogeneity, indicating the stability of the outcome data (see Figure 5).

#### 3.6.2 Meta-regression analysis

In the meta-regression analysis, the effect size served as the dependent variable, while the heterogeneous explanatory variables (covariates) included sample size, sex, intervention period, exercise intensity, exercise duration, frequency, and exercise adherence. The results indicated that only exercise adherence significantly explained the heterogeneity in 7-day point prevalence abstinence following the physical activity intervention (*p* < 0.05), whereas none of the other explanatory variables reached a significant level of explanation for the heterogeneity observed in the meta-analysis groups (*p* > 0.05).

#### 3.6.3 Subgroup analysis

The impact of exercise may be influenced by various characteristics of the exercise intervention program. A subgroup analysis was conducted to explore differences in the effect of exercise intensity (low, medium, or high), intervention duration (<8 weeks, 8 weeks, 12 weeks, or >12 weeks), and volume of exercise (<90 min/week or ≥90 min/week) on smoking cessation rates among smokers. Subgroup analyses were also performed to investigate other study characteristics that might influence the intervention effect, such as literature quality (low, medium, or high) and exercise adherence (low: <70% or high: ≥70%). The analysis employed a fixed-effects model for assessing literature quality and exercise duration, while a random-effects model was used for exercise volume and adherence. The following results were obtained (see Table 5): (World Health Organization, 2021) Regarding literature quality, the exercise intervention group exhibited higher quitting rates compared to the control group, with statistically significant differences [RR = 1.57, 95% CI (1.13, 2.18), *p* = 0.007; RR = 1.44, 95% CI (1.00, 2.06), *p* = 0.047] (Council, 2019). Concerning exercise intervention characteristics, the 8-week exercise intervention demonstrated higher 7-day point prevalence abstinence rates compared to the control group [RR = 1.54, 95% CI (1.03, 2.31), *p* = 0.036]. For the remaining intervention durations, no significant differences in quitting rates were observed between the intervention and control groups. Positive intervention effects on quitting rates were observed when the exercise volume was ≥90 min per week [RR = 1.21, 95% CI (1.03, 1.42), *p* = 0.020] (West, 2017). Only studies with high exercise adherence exhibited higher cessation rates in the intervention group compared to the control group [RR = 1.44, 95% CI (1.02, 2.02), *p* = 0.036].

**TABLE 5:** Subgroup analysis of the effect of each factor on smoking cessation rates.

Outcome	Subgroup factors	Grouping criteria	Research number	Heterogeneity test results		Effect size [95%CI]	P
				I^2^	P		
7-day point prevalence abstinence	Quality of the literature	Low	4	0	0.54	1.57 [1.13, 2.18]	0.007
		Medium	4	23.6	0.25	1.10 [0.92, 1.31]	0.295
		High	8	41.5	0.14	0.95 [0.77, 1.19]	0.668
	Duration	<8 weeks	2	45.8	0.17	1.11 [0.72, 1.70]	0.650
		8 weeks	3	44.9	0.16	1.54 [1.03, 2.31]	0.036
		12weeks	8	37.5	0.13	1.04 [0.88, 1.22]	0.673
		>12weeks	3	5.3	0.35	1.15 [0.89, 1.48]	0.295
	Volume of exercise/week	<90min	4	55.8	0.08	0.98 [0.71, 1.37]	0.919
		≥90min	9	49.6	0.05	1.21 [1.03, 1.42]	0.02
	Exercise Adherence	Low	9	0	0.56	1.05 [0.92, 1.21]	0.46
		High	6	58.8	0.03	1.44 [1.02, 2.02]	0.03
continuous abstinence	Quality of the literature	Low	3	0	0.46	1.44 [1.00, 2.06]	0.047
		Medium	4	0	0.60	0.99 [0.77, 1.27]	0.922
		High	3	0	0.44	1.04 [0.86, 1.25]	0.675

#### 3.6.4 Publication bias analysis

**FIGURE 6:**
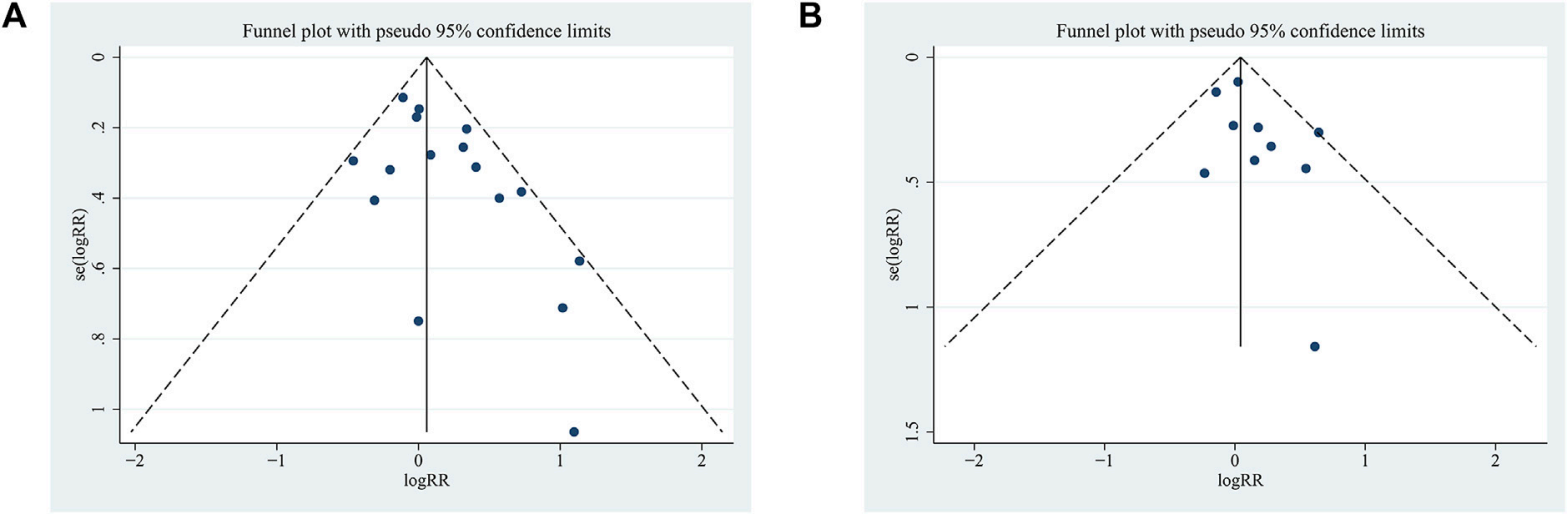
Funnel plot of the meta-analysis of a long-term exercise intervention on smoking cessation rates in tobacco-dependent individuals: **(A)** 7-day point prevalence abstinence and **(B)** continuous abstinence.

The RCTs included in the two indicators of long-term exercise amounted to 10. Ten RCTs were included for the two indicators related to long-term exercise. Therefore, a funnel plot was employed to assess publication bias. As depicted in Figure 6, the funnel plot exhibited a symmetrical distribution for the present meta-analysis. The Egger test results revealed that for 7-day point prevalence abstinence, t = −1.59, *P* > |t| = 0.137 > 0.05, and for continuous abstinence, t = −0.99, *P* > |t| = 0.349 > 0.05. These findings suggest the absence of significant publication bias.

### 3.7 Effects of exercise intervention on mood

**FIGURE 7:**
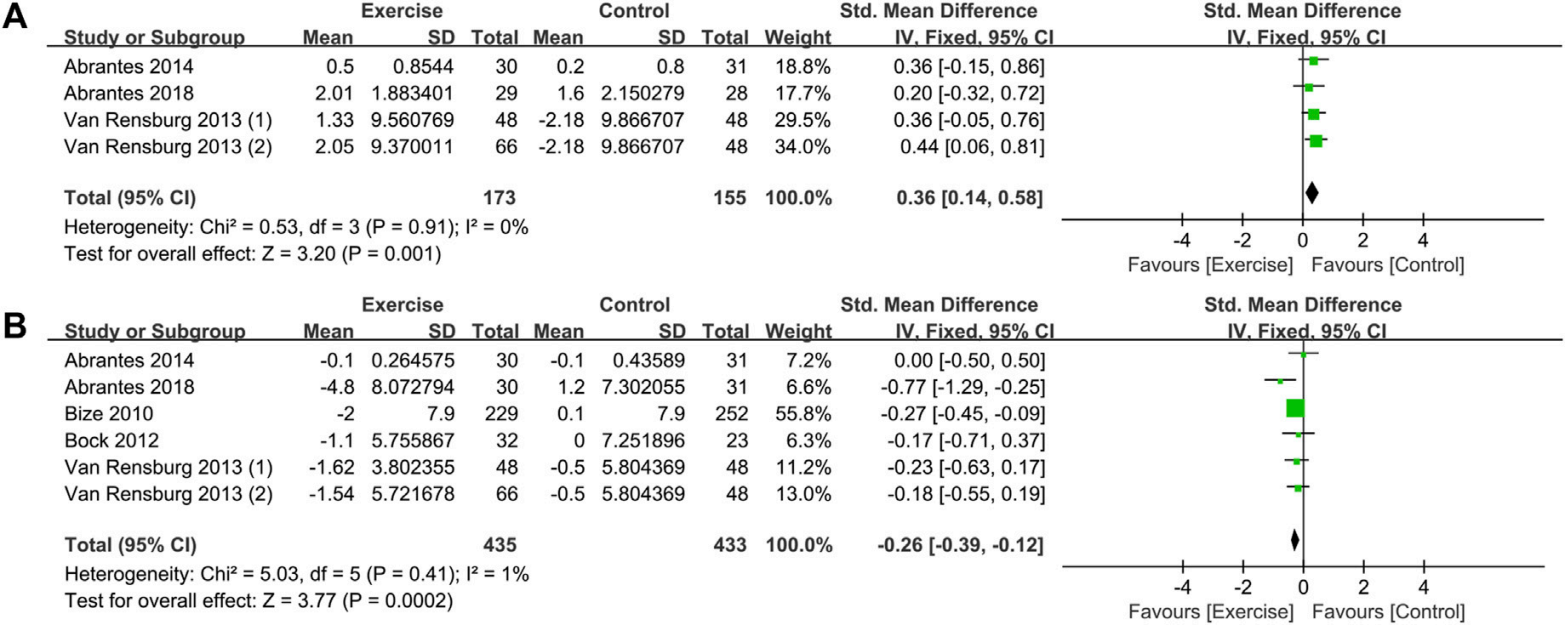
Meta-analysis of the effect of exercise on the mood of tobacco-dependent individuals: **(A)** positive mood and **(B)** negative mood.

This study provides a brief exploration of moods, specifically positive and negative moods as separate domains. The impact of exercise interventions on smokers’ mood was evaluated through six randomized controlled trials (RCTs) from five studies (Bize et al., 2010; Bock et al., 2012; Van Rensburg et al., 2013; Abrantes et al., 2014; Abrantes et al., 2018). The heterogeneity test results, as shown in Figure 7, indicated low levels of heterogeneity for both positive mood (*I* ^2^ = 0%, *p* = 0.910) and negative mood (*I* ^2^ = 1%, *p* = 0.410). Consequently, a fixed-effects model was employed for the analysis. The combined effect sizes revealed that exercise interventions effectively improved smokers’ mood, with an increase in positive mood [SMD = 0.36, 95% CI (0.14, 0.58), *p* = 0.001] and a decrease in negative mood [SMD = −0.26, 95% CI (−0.39, −0.12), *p* < 0.001]. Sensitivity analysis conducted on the included studies demonstrated the relative stability of the outcome data.

### 3.8 Quality of evidence

**FIGURE 8:**
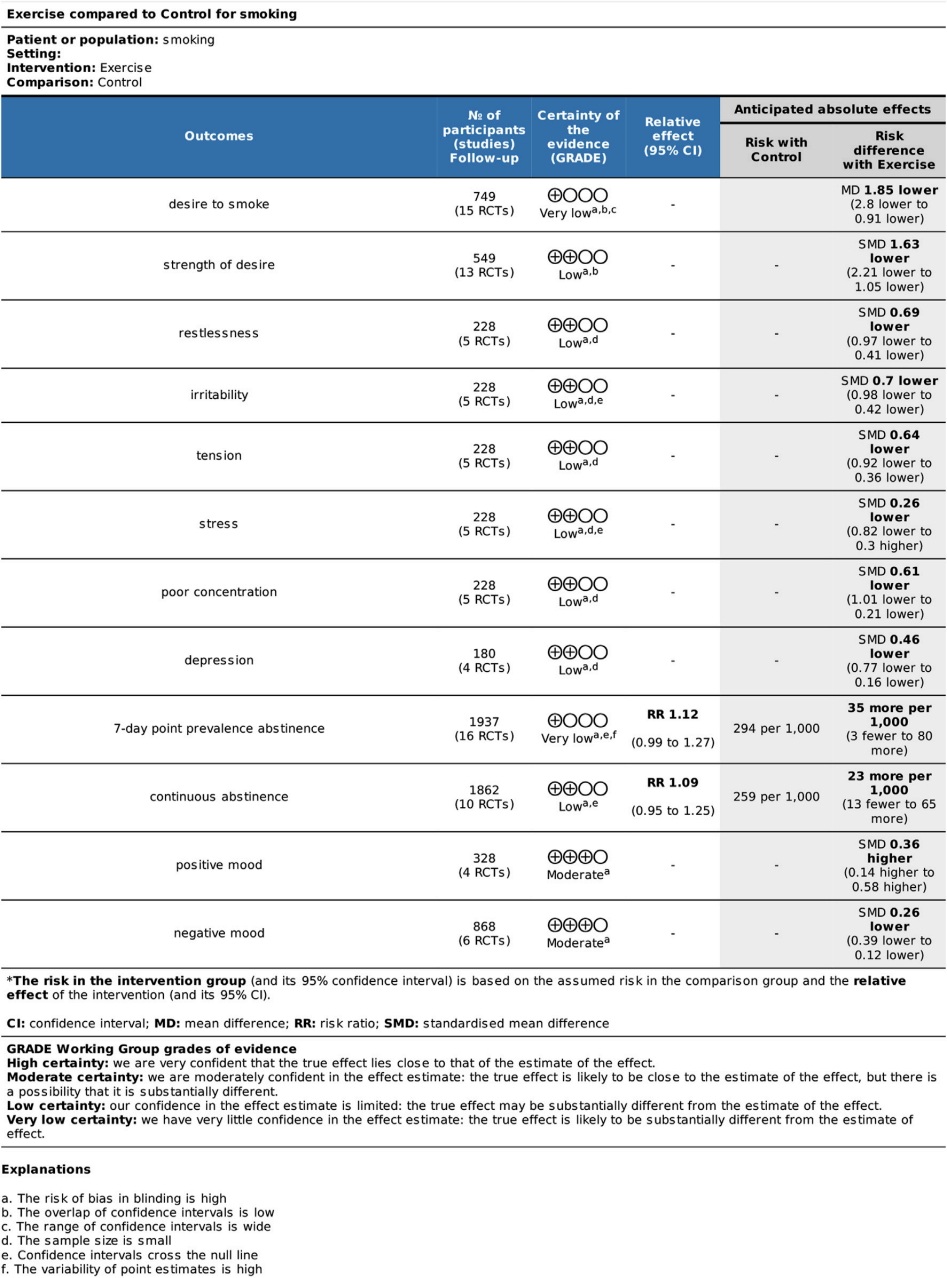
GRADE evidence profiles for exercise *versus* control.

Results of the GRADE analyses are provided in Figure 8. The outcomes DtS, and 7-day point prevalence abstinence had very low QoE; SoD, withdrawal symptoms, and continuous abstinence had low QoE; and mood had moderate QoE quality of evidence.

## 4 Discussion

The effectiveness of exercise interventions for smoking cessation has been a subject of controversy, and previous reviews have yielded mixed results. This study aimed to provide a more rigorous and comprehensive meta-analysis to evaluate the effects of exercise interventions on smoking cessation. The findings of this study indicate that short-term exercise can effectively reduce cravings for cigarettes and alleviate most withdrawal symptoms. However, long-term exercise does not appear to improve the rates of 7-day point prevalence abstinence and continuous abstinence among smokers. Additionally, exercise interventions were found to have a positive impact on mood in smokers or those attempting to quit.

### 4.1 Methodological quality assessment

Ensuring participant blinding was challenging due to the specific nature of exercise interventions, and most studies did not provide specific information on assessor blinding, resulting in a high risk of bias. Some of the included studies had missing outcome data, and although some had similar proportions of missing data across intervention groups or valid explanations, three studies still carried a high risk of bias. Furthermore, 12 studies (32%) reported other risks of bias, primarily related to small sample sizes and uneven baseline data (*p* < 0.05). Overall, the included studies were deemed to be of low quality, potentially impacting the credibility of the meta-analysis results to some extent. Subgroup analysis considering the quality of the literature as a covariate revealed that the results of low-quality literature contradicted the overall findings (i.e., long-term exercise improving smoking cessation rates *versus* long-term training not improving smoking cessation rates), indicating the influence of literature quality on the results. Future studies should strive to improve overall study quality by implementing allocation concealment, assessor blinding, increasing sample sizes, and addressing missing data issues.

### 4.2 Analysis of the effect of exercise interventions on tobacco dependence in smokers and possible mechanisms

#### 4.2.1 Craving, and withdrawal symptoms

Smoking craving and withdrawal symptoms are important indicators for assessing tobacco dependence. Most studies have utilized smoking craving to evaluate the effectiveness of acute exercise interventions for smoking cessation and have shown that a higher desire to smoke is associated with a shorter time to the next cigarette. Withdrawal symptoms experienced during the cessation process are the primary drivers of relapse, characterized by heightened negative emotions and difficulties with concentration. Consequently, reducing withdrawal symptoms is crucial for enhancing smoking cessation success rates. This study confirmed that acute exercise interventions can aid smoking cessation by reducing cravings and withdrawal symptoms.

The selection of exercise intensity is a prominent topic in the realm of acute exercise interventions for smoking cessation. The subgroup analysis conducted in this study revealed that low, medium, and high-intensity exercise all alleviated cravings and withdrawal symptoms in smokers. However, low-intensity exercise was not found to be effective in reducing symptoms of depression. Previous studies comparing the effects of different exercise intensities on craving interventions have shown no significant difference in craving changes between medium- and high-intensity exercise at the end of the intervention. However, high-intensity exercise exhibited certain advantages, including a longer-lasting intervention effect and preventing smokers from shifting their attention to smoking-related cues (attentional bias) (Everson et al., 2008; Scerbo et al., 2010; Oh and Taylor, 2014). Conversely, Robert et al. found that, compared to moderate-intensity exercise, high-intensity exercise can significantly reduce the desire for cigarettes in the short term (Roberts et al., 2015). The findings of this study suggest that this discrepancy may be attributed to the characteristics of the study sample, which included individuals with a high level of tobacco dependence. This implies that high-intensity exercise may have a more pronounced effect on reducing cravings than moderate-intensity exercise in smokers with high levels of tobacco dependence.

These findings are consistent with the results of the present meta-analysis, which demonstrated a strong association between the level of tobacco dependence and the effectiveness of acute exercise interventions. Smokers with high levels of tobacco dependence were less likely to benefit from exercise. In conclusion, for tobacco-dependent patients, acute exercise interventions should primarily consist of moderate intensity, taking into account the degree of dependence. When the level of dependence is high, appropriately increasing the intensity could lead to better intervention effects.

Exercise interventions may impact cravings in smokers through the following mechanisms:

Cognitive improvement: Exercise plays a role in modulating attentional bias, increasing cognitive load on the brain’s information processing capacity, and reducing activation in brain regions associated with reward processing and visuospatial attention (Van Rensburg et al., 2009b). Additionally, exercise increases activation in brain regions of the medial prefrontal cortex related to the brain’s default mode, shifting attention away from tobacco-related cues (Van Rensburg et al., 2012). Moreover, brief periods of exercise induce physiological changes that cause structural (increase in white matter and gray matter volumes) (Suo et al., 2016; Bashir et al., 2021) and functional (cerebral metabolism and cerebral blood flow) (Renke et al., 2022) alterations in the brain. These changes promote the remodeling of brain structures like the prefrontal lobe and striatum, enhance inhibitory control in tobacco-dependent individuals, and consequently reduce tobacco dependence.

Reward substitution: Exercise effectively increases the levels of substances such as endorphins in tobacco-dependent individuals, compensating for the rewarding pleasure derived from smoking through neurohumoral regulation (Georgakouli et al., 2020). Furthermore, exercise enhances dopaminergic activity in the limbic reward system, improves the function of the midbrain dopaminergic system, and stabilizes the structure of the midbrain dopamine system. This normalization of reward and treatment pathways in the brains of tobacco-dependent patients helps overcome the lack of euphoria caused by nicotine withdrawal, thereby enhancing their psychological level of pleasure and promoting positive emotional experiences (Georgakouli et al., 2020).

#### 4.2.2 Smoking cessation rate

A total of 19 of the included studies addressed the effect of long-term exercise interventions on tobacco dependence in smokers. Only three of the 17 studies reported a positive effect of exercise on smoking cessation (Marcus et al., 1991; Marcus et al., 1999; Bock et al., 2012). The results of meta-analysis showed that no significant difference in quit rates between the exercise and control groups in long-term exercise interventions, which is generally consistent with previous results (Ussher et al., 2019).

Exercise adherence and frequency are potential factors influencing the effectiveness of long-term exercise interventions. The majority of in-home exercise studies have shown poor exercise adherence. Kinnunen’s study (Kinnunen et al., 2008) revealed that less than 50% of participants followed the prescribed exercise regimen during the initial 5 weeks, and this percentage dropped to 6.5% by the end of the treatment period. Marcus (Marcus et al., 2005) found that only 15.2% of participants adhered to the exercise prescription. A study investigating the relationship between exercise effects and adherence discovered a moderate association between higher exercise frequency and improved 7-day quit rates as well as longer quit times during and after treatment (Williams et al., 2010). Consequently, the positive impact of exercise may be compromised by a lack of strict adherence to the exercise prescription. A subgroup analysis based on exercise adherence in this study indicated that the exercise group with high adherence demonstrated a higher 7-day point quitting rate. However, a more recent study in the higher quality literature revealed that despite approximately 85% of sessions being attended across different treatment conditions and 88% of exercise sessions being completed within the prescribed moderate intensity range, this level of compliance was insufficient to demonstrate an improvement in smoking cessation rates through exercise intervention (Dunsiger et al., 2021). Considering that the intervention effect of acute exercise on craving gradually diminishes after approximately 30 min, Williams’ study showed that exercising three times per week resulted in favorable acute changes in affect and cigarette cravings from pre-to post-exercise, but exercise did not consistently influence affect or craving on a session-to-session basis. Dunsiger (Dunsiger et al., 2021) also implemented an intervention protocol involving exercise three times per week with extended intervals between interventions. Long-term exercise at lower intervention frequencies may not sustainably reduce cravings and withdrawal symptoms, necessitating frequent and consistent exercise over time to achieve benefits. Therefore, future studies could investigate whether ensuring both frequency and exercise compliance in long-term exercise interventions could enhance smoking cessation outcomes.

#### 4.2.3 Mood

The study findings indicate that exercise has a greater impact on mood improvement, encompassing both an increase in positive mood and a decrease in negative mood. However, upon reviewing the literature, we identified a lack of differentiation between “emotion,” “affect,” and “mood” in current research on exercise interventions for smoking cessation. These three concepts were often conflated, which was also evident in the literature used as an outcome indicator in our study. Additionally, referencing the relevant literature by Ekkekakis (Ekkekakis, 2003; Ekkekakis, 2013), and a study on PANAS (Watson et al., 1988), one of the selected emotion rating scales, it became evident that the scale itself has certain limitations. Despite being described as a mood rating scale by its developers, the scale’s name suggests an affect rating scale, and the internal items encompass both mood, emotions, and affect. It is important to emphasize that “mood,” “emotion,” and “affect” are distinct terms and cannot be used interchangeably. Therefore, it is recommended that future studies make clear distinctions and provide a comprehensive understanding of the research framework.

## 5 Limitations

The study has several limitations that may impact the reliability of the findings. These limitations include difficulties in contacting authors of some literature, lack of standardization in physical activity variables (e.g., frequency, intensity, duration, and type), and potential confounding factors such as adherence. Firstly, the inability to reach authors for specific literature sources hinders data verification. Additionally, due to the nature of exercise interventions, achieving blinding is challenging, and most studies inadequately describe whether assessor blinding and allocation concealment were implemented, resulting in increased methodological heterogeneity. Secondly, during data processing, it was observed that there was considerable heterogeneity among the studies that used craving as an outcome indicator. However, after conducting regression and subgroup analyses to explore potential contributing factors to heterogeneity, no specific source of heterogeneity could be identified. Lastly, although cardiopulmonary function and mood are presumed to be important regulatory variables in the relationship between exercise and tobacco dependence, only a limited number of studies have provided clarification on the participants’ level of cardiorespiratory function and mood status, which prevented their data from being included in this study.

## 6 Conclusion

Based on this meta-analysis, it is evident that acute exercise significantly reduces cravings and withdrawal symptoms in smokers, thus supporting its potential role in smoking cessation. However, the effectiveness of long-term exercise interventions remains inconclusive, as long-term exercise did not yield higher quit rates. Exercise can help reduce negative mood and enhance positive mood in smokers. Therefore, future research of higher quality is required. Furthermore, greater attention should be given to strategies aimed at improving exercise adherence in long-term interventions, as well as reevaluating the intervention effects by reducing the interval between interventions. Additionally, it is recommended that future studies accurately differentiate between mood, emotion, and affect.

## Data Availability

The original contributions presented in the study are included in the article/Supplementary Material, further inquiries can be directed to the corresponding author.
